# Factor XII in Thrombosis and Thromboinflammation: From Molecular Biology to Clinical Translation

**DOI:** 10.3390/ijms27073336

**Published:** 2026-04-07

**Authors:** Jan Stępnicki, Anna M. Imiela, Marta Szymańska, Jakub Mikołajczuk, Piotr Pruszczyk

**Affiliations:** Department of Internal Medicine & Cardiology with the Centre for Management of Venous Thromboembolic Disease, Medical University of Warsaw, 02-005 Warsaw, Poland; anna.imiela@uckwum.pl (A.M.I.); piotr.pruszczyk@wum.edu.pl (P.P.)

**Keywords:** factor XII, stroke, thrombosis, thromboinflammation, HAE

## Abstract

Factor XII (FXII) is a central mediator at the intersection of coagulation, fibrinolysis, inflammation, and immunity. It is activated upon contact with negatively charged surfaces, triggering the intrinsic coagulation pathway and driving thrombus formation and stabilization. Beyond clotting, FXII contributes to activation of the kallikrein–kinin system, generation of bradykinin, and modulation of inflammatory and immune responses. Congenital FXII deficiency does not increase bleeding risk, highlighting its unique role and making FXII inhibition an attractive strategy for anticoagulation and immune modulation with a potentially superior safety profile. Preclinical studies provide compelling evidence for this concept. In models of ischemic stroke and traumatic brain injury, FXII blockade significantly reduced infarct volume, improved neurological outcomes, and attenuated neuroinflammation without increasing hemorrhage. Similarly, in extracorporeal circulation and vascular stent implantation, FXII inhibition prevented thrombus formation and reduced fibrin deposition, achieving effects comparable to heparin but with markedly lower bleeding risk. Several classes of FXII inhibitors are currently in development, including antisense oligonucleotides, peptides, recombinant proteins, and monoclonal antibodies. Among them, Ixodes ricinus contact phase inhibitor (Ir-CPI) and recombinant human albumin-fused Infestin-4 (rHA-Infestin-4) have demonstrated strong antithrombotic efficacy in animal models. Most notably, garadacimab, a monoclonal anti-FXIIa antibody, has completed phase 3 trials and received regulatory approval for hereditary angioedema (HAE) prophylaxis, where it markedly reduces attack frequency with a favorable safety profile. This review summarizes current knowledge on FXII biology and evaluates its translational potential as a novel target for anticoagulant and anti-inflammatory therapies.

## 1. Introduction

As our understanding of human molecular biology deepens, factor XII (FXII) has emerged as a promising target in the treatment of various diseases. FXII plays a central role in human physiology, linking several critical pathways: coagulation, fibrinolysis, inflammation, and immunity [1]. Consequently, its inhibition presents a novel strategy for anticoagulant therapy and immune modulation.

Notably, individuals with congenital FXII deficiency do not exhibit an increased risk of bleeding [2,3], as physiological hemostasis is predominantly initiated via the tissue factor-driven extrinsic pathway, rendering the contact pathway largely redundant under normal conditions. FXII appears as an attractive target in cardiovascular disease, particularly where coagulation balance is disrupted, leading to hypercoagulability or hemorrhage. FXII is auto-activated upon contact with negatively charged surfaces and initiates the intrinsic coagulation pathway, subsequently promoting thrombus formation and stabilization [4]. This mechanism is especially relevant in the context of thrombosis associated with extracorporeal circuits, endovascular procedures, and stent implantation.

This review summarizes current insights into the therapeutic potential of FXII inhibitors, with particular focus on their role in modulating the intrinsic pathway and preventing ischemic stroke. Additionally, it examines the relation between FXII and complement system, the impact of FXII inhibition on the kallikrein–kinin system, and its clinical application in the treatment of hereditary angioedema (HAE). Finally, we evaluate the advantages and limitations of FXII inhibitors within the broader landscape of antithrombotic therapies, and highlight key compounds currently undergoing clinical trials.

## 2. Methods

Among the available databases, we selected Pubmed (National Library of Medicine, NIH; pubmed.ncbi.nlm.nih.gov) to search for articles related to the topic of the current review using the keywords “factor XII inhibitors”. Articles published between 1950 and 2025 were included in further analysis with particular attention to recently published studies. They were classified thematically into those related to immunomodulation or coagulation, and subsequently thromboprophylaxis and ischemic stroke management. Animal model and human studies were further divided into subgroups. Results were compared to find common conclusions and discrepancies with a focus on their potential clinical applicability. In addition, results were compared in tabular form to find common conclusions and chronologically outline the progress of research. Subsequently we again screened Pubmed for current information on clinically most relevant factor XII inhibitors using keywords: “Ir-CPI”, “rHA-Infestin-4”, and “FXII monoclonal antibodies inhibitors”.

Exclusion criteria comprised duplicate publications; articles not primarily focused on factor XII or factor XII inhibitors; studies not addressing coagulation, thrombosis, thromboinflammation, ischemic stroke, extracorporeal circulation, hereditary angioedema, or related translational implications; conference abstracts, editorials, commentaries, letters without substantial original or mechanistic data; and articles for which full text was unavailable.

## 3. FXII Inhibitors in Anticoagulant Pharmacotherapy: A Historical Perspective

The history of anticoagulant therapy dates back to the early 20th century. Since the discovery of unfractionated heparin (UFH) in 1916 [5] and its first use in humans in the 1940s, there has been an ongoing search for drugs that provide both effective and safe anticoagulation.

Heparins exert their effect by binding to and activating antithrombin, thereby inhibiting thrombin (FIIa) and factor Xa (FXa), among other targets [6]. A major advancement occurred in the late 1980s and early 1990s with the development of low-molecular-weight heparin (LMWH). This form offered several advantages over UFH, including subcutaneous administration, a longer half-life, reduced risk of heparin-induced thrombocytopenia, and consequently, a lower bleeding risk [7]. Fondaparinux, a synthetic pentasaccharide introduced in the early 2000s, selectively binds FXa without affecting thrombin, offering an improved safety profile [8,9].

Vitamin K antagonists (VKAs) are characterized by a different mechanism of action; they disrupt vitamin K metabolism, thereby preventing the production of clotting factors II, VII, IX, and X [10]. Warfarin, a commonly used VKA, was introduced for clinical use in the 1960s. Despite their narrow therapeutic index and variable pharmacokinetics and pharmacodynamics, VKAs remain the preferred anticoagulants in specific clinical contexts, including patients with mechanical heart valves, significant mitral stenosis, or antiphospholipid syndrome (APS) following thromboembolic events [11,12].

The introduction of direct oral anticoagulants (DOACs) in the last decade marked another breakthrough in anticoagulant therapy. Direct thrombin inhibition (dabigatran) and factor Xa inhibitors—rivaroxaban, apixaban, and edoxaban—transformed anticoagulant therapy by offering fixed-dose regimens, oral administration, and elimination of the need for regular laboratory monitoring. These drugs have shown efficacy comparable to the VKA therapies in preventing thromboembolic complications in atrial fibrillation and in the management of venous thromboembolism (VTE) [13,14]. Nevertheless, despite the significant progress brought by DOACs, their use is still associated with adverse effects, including gastrointestinal and intracranial bleeding.

It is therefore not surprising that the search for new anticoagulant targets continues, with the goal of achieving an even better safety profile. Factor XII (FXII) was initially identified in 1955 in the preoperative blood sample of a 37-year-old patient, John Hageman. A prolonged clotting time was observed, despite the absence of hemorrhagic symptoms. Blood samples from his relatives revealed an autosomal recessive deficiency, and the factor was subsequently named “Hageman factor” [2]. Interest in FXII has grown alongside advances in human biochemistry. Understanding the diverse functions of FXII in physiological processes—such as the intrinsic coagulation cascade, the kallikrein–kinin pathway, and immune regulation [15]—has prompted exploration of FXII inhibitors as promising candidates for safe anticoagulation and possible immunomodulatory therapies, as outlined in the next sections.

## 4. Molecular Biology of FXII

### 4.1. FXII in Coagulation Cascade–Intrinsic Pathway and Crosstalk with Tissue Factor Pathway

Factor XII (FXII) is a zymogen, a glycoprotein precursor of approximately 90 kDa, that becomes the active enzyme FXIIa upon proteolytic activation.

FXII is classically activated upon contact with negatively charged surfaces, including damaged vascular endothelium, extracellular matrix components, neutrophil extracellular traps (NETs), and polyphosphates released from activated cells or platelets [16,17]. This reaction underlies the activated partial thromboplastin time (aPTT) test, also known as the kaolin–cephalin clotting time (KCCT), where kaolin serves as a well-characterized activator of FXII-dependent coagulation [18].

**Figure 1:**
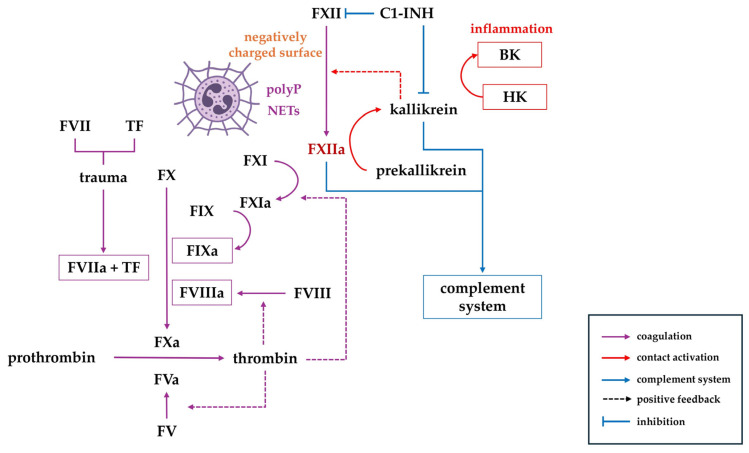
Factor XII (FXII) is a central initiator of the intrinsic coagulation pathway and plasma contact system. Upon exposure to negatively charged surfaces, polyphosphates released from damaged cells or neutrophil extracellular traps (NETs); FXII is activated to FXIIa, which triggers FXI-dependent thrombin generation. Tissue factor (TF)–FVIIa complex starts the extrinsic pathway and generates small amounts of thrombin, which feeds back to activate FXI and amplifies the FXII/FXI-driven intrinsic pathway [33]. Concurrently, FXIIa initiates a contact activation system via plasma kallikrein, which further generates bradykinin (BK) by cleavage of high-molecular-weight kininogen (HK). The kallikrein–FXII axis operates as a self-amplifying positive feedback loop, in which FXIIa generates plasma kallikrein, which in turn further activates FXII, thereby propagating contact system activation and thromboinflammatory signaling. Plasma kallikrein and FXIIa are capable of activating the complement cascade. C1 inhibitor (C1-INH) serves as a key regulator of both the complement and contact systems, including FXIIa and kallikrein.

Once activated, FXIIa initiates the intrinsic coagulation pathway by activating factor XI (FXI). FXIa subsequently activates factor IX (FIX), which, in combination with its cofactor FVIIIa, forms the tenase complex that converts factor X (FX) into its active form, FXa. FXIIa additionally contributes to fibrinolysis, both directly and through its interaction with prekallikrein (PK), promoting plasmin generation from plasminogen (Figure 1) [19].

Although historically viewed as distinct initiation mechanisms, accumulating evidence shows that the tissue factor (TF)-initiated extrinsic pathway and the contact-mediated intrinsic pathway converge and reinforce each other during physiological and pathological coagulation.

At a biochemical level, TF exposed at sites of vascular injury or expressed by activated leukocytes engages circulating FVIIa, forming the TF:FVIIa complex that rapidly generates FXa and small amounts of thrombin. It feeds back into intrinsic components of coagulation by activating platelets and several intrinsic factors, notably FVIII, FV, and FXI [20]. It enables the intrinsic tenase (FIXa:FVIIIa) to propagate thrombin formation even in the absence of strong contact activation surfaces. Mathematical models and in vitro data support the quantitative contribution of thrombin-mediated FXI activation to overall thrombin generation during TF-initiated coagulation [20]. Looking at the relationship between FXII and TF from the other side, contact-induced FXIIa facilitates FXI activation and kallikrein generation, which in turn can augment inflammation and provide additional procoagulant surfaces (e.g., NETs). This reinforces coagulation that is initially triggered via TF.

### 4.2. FXII in Inflammation-Contact Activation System and Its Relation to Complement System

Accumulating evidence over recent years has highlighted the pivotal role of the immune system in thrombosis. Thrombosis potentiates inflammation, whereas excessive inflammatory responses promote thrombus formation, largely via activation of platelets, leukocytes, and endothelial cells [21]. Platelet activation during thrombus formation results in the release of potent vasoconstrictors, including thromboxane A_2_, adenosine diphosphate, prostaglandins, and serotonin [22]. Endothelial cells constitute a mechanical barrier between smooth muscle cells (SMCs) and circulating blood, serving as a central regulator of inflammatory responses and vascular permeability [23].

Although the complement and contact systems were traditionally considered distinct plasma proteolytic pathways, accumulating evidence now demonstrates extensive biochemical and functional crosstalk between factor XII (FXII), the contact system, and the complement cascade [24].

FXIIa cleaves PK into its active form, kallikrein, which in turn amplifies FXII activation in a positive feedback loop [25]. Kallikrein subsequently cleaves high-molecular-weight kininogen (HK) to generate bradykinin (BK), a mediator of vasodilation and neutrophil recruitment [26,27]. Bradykinin engagement of the B2 receptor triggers intracellular calcium mobilization and downstream production of nitric oxide, eicosanoids, and tissue-type plasminogen activator. This signaling axis increases vascular permeability, promotes nitric oxide-dependent vasodilation and hypotension, and amplifies inflammatory responses, including edema and pain, while further supporting leukocyte recruitment via macrophage-derived chemotactic mediators and direct neutrophil activation [28,29]. Together, FXII, PK, and HK constitute the plasma contact system, linking coagulation with inflammation and vascular permeability. Plasma kallikrein can directly cleave complement factors C3 and C5, producing the anaphylatoxins C3a and C5a through mechanisms that bypass the classical complement cascade. Moreover, FXIIa and its β-FXIIa fragment have been shown to interact with components of the C1 complex (C1q–C1r–C1s), thereby triggering classical complement activation in vitro. Biochemical studies identified FXIIa-derived fragments capable of engaging the C1 complex [30]. Collectively, these interactions establish a mechanistic link between the contact system and the complement cascade at the level of proteolytic activation. Shared protease inhibitors, most notably C1-esterase inhibitor (C1-INH), regulate both the complement and contact systems. C1-INH suppresses key complement proteases (C1r, C1s, and MBL-associated serine proteases-MASPs) as well as FXIIa and plasma kallikrein, thereby limiting activation across both cascades. Conversely, quantitative or functional C1-INH deficiency, as observed in hereditary angioedema (HAE), results in dysregulated activation of the contact/kallikrein–kinin system and complement proteases [31,32]. These interconnected pathways explain why FXII inhibition influences not only thrombosis but also inflammation and vascular leakage, a concept central to FXIIa-targeting therapeutics in HAE, such as garadacimab.

## 5. FXII’s Contribution to Thrombosis: A Shift in Understanding

As noted earlier, FXII was discovered more than 70 years ago. But what has reignited interest in its role within the coagulation cascade and prompted recent investigations into FXII inhibitors as potential antithrombotic agents? One key factor was the growing recognition of the contact system’s role in thrombus formation and stabilization. Pivotal animal studies published in the early 2000s have demonstrated that mice deficient in FXII or FXI formed smaller and less stable clots at sites of arterial or venous injury [34,35]. Furthermore, introducing human FXII into FXII-deficient mice corrected their extended aPTT and restored normal thrombotic responses. These findings challenged the long-standing belief that the FXII-initiated intrinsic pathway was irrelevant for clotting in vivo. On the contrary, FXII proved to be crucial—and perhaps even evolutionarily significant.

A particularly compelling observation was the role of FXII in the hemostasis of contaminated wounds. In wild-type mice, exposure to silicon found in soil has significantly reduced blood loss compared to FXII−/− mice. Soil was shown to accelerate plasma clotting in humans and mice through an FXII-dependent mechanism, but this effect was absent in cetaceans and birds, which naturally lack FXII [36]. These observations have suggested that the extrinsic and intrinsic pathways play complementary roles: FVII deficiency delays the initiation of clotting, whereas FXII deficiency results in unstable clot formation [37].

Historically, it was thought that only negatively charged surfaces—such as those used in extracorporeal circuits for cardiopulmonary bypass or hemodialysis—could activate the contact system [38]. However, major advances have come with the discovery of naturally occurring substances, which can also activate FXII. Brill et al. have revealed that polyphosphates (polyP) released from injured cells, as well as neutrophil extracellular traps (NETs), trigger coagulation through FXII-dependent mechanisms [17]. In a fascinating evolutionary twist, sand fly saliva was found to contain proteins that act as anticoagulants by targeting this mechanism [39]. These proteins bind polyP with high affinity, inhibiting FXII and FXI autoactivation and thus suppressing the intrinsic pathway. Interestingly, long-chain polyP can also promote FXII-mediated coagulation even in the absence of FXI activity, suggesting an alternative pathway of procoagulant activation [40]. Collectively, these findings have shifted the perception of FXII from a redundant factor to a promising target in thrombosis research and therapy.

### FXII in Cancer-Driven Thrombosis

It is important to note that exposure of the contact system to polyphosphates (polyPs) can also occur in pathological conditions—most notably in tumors that secrete them. In murine models, prostate cancer cells were shown to initiate coagulation through an FXII-dependent mechanism via long-chain polyPs expressed on their surface [41]. This procoagulant effect was markedly reduced when FXII or FXI was absent. In addition, genetic deficiencies of FXI, FXII, or high-molecular-weight kininogen (HK) safeguarded mice against cancer-associated fatal pulmonary embolism (PE).

Further studies have demonstrated that targeting the polyphosphate–factor XII (polyP–FXII) axis can attenuate cancer-associated thrombosis in preclinical models, without increasing bleeding risk [41]. These findings suggest a potential avenue for anticoagulant therapy tailored to specific cancer types. However, current data on the effectiveness of FXII inhibitors across various oncological settings has remained limited.

Taken together, these results support the hypothesis that FXII inhibition could effectively reduce the risk of thromboembolic complications—particularly those driven by activation of the intrinsic pathway.

## 6. FXII in the Pathophysiology of Ischemic Stroke and Brain Trauma

Factor XII (FXII) plays a role in the pathophysiology of brain injury via two primary mechanisms. First, activated FXII (FXIIa) initiates the intrinsic coagulation pathway, promoting thrombus formation in cerebral vessels. Second, FXIIa activates the kallikrein–kinin system, leading to the release of bradykinin (BK), a potent proinflammatory mediator. These processes have been studied in animal models (Table 1).

The transient middle cerebral artery occlusion (tMCAO) model is commonly used to simulate ischemic stroke and assess ischemia–reperfusion injury in the brain. In a study by Kleinschnitz et al. [42], tMCAO was performed on two groups of mice: wild-type and FXII-deficient. Mice lacking FXII exhibited more than a 50% reduction in infarct volume within 24 h after reperfusion compared to wild-type controls. Furthermore, FXII-deficient mice performed better on the Bederson score [43] and grip strength test, which assess global neurological and motor function, respectively. However, when human FXII was intravenously administered to the FXII-deficient mice, the protective effect was reversed, resulting in infarct sizes and neurological scores comparable to wild-type mice. These observations were confirmed by Wang et al. in their work using a peptide inhibitor of FXIIa and plasma kallikrein (PKa)-CycloSD6 [44,45,46]. In an ischemic stroke model, mice treated with it showed better neurological function. Furthermore, analysis of brain tissue revealed that CycloSD6 significantly reduced ischemia–reperfusion injury and preserved blood–brain barrier integrity.

In addition to its role in stroke, FXII has been implicated in the pathology of traumatic brain injury (TBI). In a study by Hopp et al. [47], wild-type mice subjected to experimental closed-head injury developed a greater number of occluded cerebral microvessels compared to FXII-deficient mice. Treatment with an FXII inhibitor improved cerebral blood flow, reduced neurodegeneration, and restored motor function in wild-type mice. Similarly, when FXII-deficient mice were reconstituted with human FXII, they became more susceptible to injury. Furthermore, in a cortical cryolesion model, FXII-deficient mice demonstrated a more rapid reduction in lesion volume compared to wild-type controls. These findings suggest that FXII plays a detrimental role in thromboinflammatory processes but its inhibition may provide a neuroprotective effect in both ischemic stroke and traumatic brain injury.

**Table 1:** Summary of selected studies evaluating the role of factor XII in the experimental animal models of ischemic stroke.

Author	Year	Species and FXII Inhibitors	Main Conclusion
Kleinschnitz C et al. [42]	2006	○ wild-type mice ○ FXII-deficient mice	FXII^−/−^ mice are protected from cerebral ischemia, showing > 50% reduction in infarct volume 24 h after tMCAO compared with wild-type controls. Neurological outcomes are significantly improved in FXII-deficient animals. Intravenous reconstitution with human FXII restores susceptibility to ischemic brain injury. FXII deficiency does not increase intracranial hemorrhage following tMCAO.
Hagedorn I et al. [48]	2010	○ wild-type rats and mice ○ NMRI mice ○ CD rats ○ C57Bl/6 mice ○ rHA-Infestin-4	rHA-Infestin-4 completely abolished FeCl3-inducedocclusive arterial thrombus formation in mice and rats while leaving hemostasis fully intact. rHA-Infestin-4 was highly protective in a murine model of ischemic stroke.
Chen JW et al. [49]	2012	○ Balb/c mice ○ injection of fluorescently-labeled microbeads or fractionated clot ○ MRI assessment ○ rHA-Infestin-4	rHA-Infestin-4 administration significantly reduced ischemic damage and pathological coagulation without increasing hemorrhagic frequency in mice after induced silent brain ischemia (SBI).
Krupka J et al. [50]	2016	○ male CD rats ○ rHA-Infestin-4	Within prophylactic treatment with rHA-Infestin-4 before tMCAO, infarct areas and brain edema formation were reduced accompanied by better neurological scores and survival compared to controls. Following therapeutic treatment (after the start of reperfusion), neurological outcome and survival were still improved although overall effects were less pronounced compared to prophylaxis.
Hopp S et al. [47]	2016	○ wild-type mice ○ FXII-deficient mice ○ FXI-deficient mice ○ experimental closed-head injury	Genetic FXII deficiency or pharmacologic FXIIa inhibition reduces trauma-induced microvascular thrombosis in mice. This is associated with improved neurological function, smaller lesion volumes, and attenuated neurodegeneration. Reconstitution with human FXII restores thrombus formation and brain injury in FXII-deficient animals.
May F et al. [51]	2016	○ NMRI mice ○ C57Bl/6 mice ○ New Zealand White rabbits ○ Chinchilla Bastard rabbits ○ rHA-Infestin-4	rHA-Infestin-4 reduces thrombotic occlusion in mechanically induced arterial and FeCl_3_-driven venous thrombosis models. It protects against arterial and stasis-induced venous thrombosis in rabbits. rHA-Infestin-4 prevents foreign surface-triggered thrombosis in arteriovenous shunt models (mice and rabbits). It preserves hemostasis, with no detectable impairment of bleeding parameters in rabbits.
Wang W et al. [46]	2025	○ C57BL/6J mice ○ Balb/c mice	CycloSD6, a cyclized derivative of the short peptide SD6: exhibits enhanced inhibition of FXIIa and plasma kallikrein. It also demonstrates robust antithrombotic efficacy in multiple murine models (carrageenan-induced tail thrombosis, FeCl_3_ arterial thrombosis, cortical photothrombosis). CycloSD6 does not prolong bleeding time, indicating preserved hemostatic function.

tMCAO—transient middle cerebral artery occlusion; NMRI—Naval Medical Research Institute; rHA-Infestin-4—recombinant human albumin-fused Infestin-4; MRI—magnetic resonance imaging.

## 7. Targeting FXII to Mitigate Thrombogenicity of Artificial Surfaces

Due to the way FXII is activated upon exposure to polyanionic artificial materials [41], its inhibition offers promising potential for peri-procedural anticoagulation, particularly during the use of vascular catheters and extracorporeal circuits. It also seems to be worth considering in need of chronic anticoagulation in selected groups of patients after vascular grafts implantation.

One of the primary limitations of such procedures is thrombogenicity and immune activation, especially in critically ill patients who already exhibit heightened inflammatory responses and a simultaneous risk of both bleeding and thrombosis [52]. As a central link between the coagulation cascade and the kallikrein–kinin system, FXII has become a focal point for reducing these complications, which are the challenge with use of current anticoagulation strategies. In FXII-deficient rodents exposed to extracorporeal membrane oxygenation (ECMO), inflammatory markers, including neutrophil infiltration of target organs, were significantly reduced compared with wild-type controls, even under pharmacologic control of coagulation [53]. Moreover, FXII appeared to promote inflammation and organ injury through mechanisms independent of thrombin, as FXII knockout or inhibition reduced neutrophil recruitment and kininogen cleavage; nonetheless, in settings of increased tissue factor, thrombin-dependent FXI activation may dominate the coagulation response, masking FXII-specific contributions [54].

Vascular access catheters and filtration membranes used in hemodialysis or ECMO are typically made of synthetic polymers. These materials, due to their negatively charged surfaces, activate FXII and initiate the intrinsic coagulation pathway [55]. Conventional polymer membranes still present challenges such as limited biocompatibility, permeability, fouling resistance, and thrombogenicity—driving research into biomimetic membranes inspired by endothelial surfaces. Although promising, these membranes are not yet widely implemented [56], creating an urgent need for safe pharmacological anticoagulation strategies.

Polyvinyl chloride (PVC), a common synthetic polymer, is a well-known activator of FXII. Contact between blood and PVC induces robust thrombin generation, which can be effectively suppressed through pre-treatment with anti-FXII antibodies [57]. However, coagulation activation in this setting cannot be reliably monitored through levels of free FXIIa or FXIIa–C1-inhibitor complexes, as these remain unchanged post-thrombin generation.

Improving blood flow and maintaining vessel patency through the use of stents and stent grafts contributes significantly to both increased life expectancy and symptom relief in patients with various vascular diseases—most commonly those caused by atherosclerosis. The development of antiplatelet and anticoagulant therapies remains a cornerstone of modern cardiology and vascular surgery. However, these therapies are not without risks. Bleeding, including life-threatening hemorrhage, remains a serious complication. Conversely, some patients—despite receiving guideline-directed treatment—still experience stent thrombosis. This ongoing challenge underscores the need for safer and more effective anticoagulant strategies.

A range of FXII inhibitors, monoclonal antibodies and proteins such as Ixodes ricinus contact phase inhibitor (Ir-CPI), have been studied in animal models extracorporeal circuit and arteriovenous shunts, as alternatives or complements to the current standard of treatment (Table 2).

## 8. FXII Inhibitors and Their Characteristics

Given the multifaceted role of FXII in coagulation, fibrinolysis, inflammation, and immunity, a wide range of anti-FXII(a) agents have been developed—each differing in their pharmacokinetic and pharmacodynamic profiles. These include small molecules, peptides, proteins, monoclonal antibodies, antisense oligonucleotides (ASOs), and small interfering RNAs (siRNAs). The various classes differ in target specificity, duration of action, administration route, side effect profiles, and the potential for rapid reversal.

ASOs and monoclonal antibodies typically require intravenous administration [66,67], while small molecules offer potential for development into oral formulations. ASOs act by suppressing hepatic synthesis of FXII, but their therapeutic effects develop slowly over several weeks. In contrast, antibodies and certain proteins can block FXII activation or downstream interactions almost immediately after administration. Although monoclonal antibodies offer high selectivity, they may also activate the immune system, leading to long-term immunogenicity-related complications.

For the purpose of this review, we have focused primarily on FXII inhibitors, which currently seem to represent the greatest clinical relevance: Ir-CPI, rHA-Infestin-4 and the monoclonal antibody garadacimab. Monoclonal antibodies were also included due to their high therapeutic potential in animal model studies, although their clinical implications seem limited due to the aforementioned characteristics (Table 3).

### 8.1. Ixodes Ricinus Contact Phase Inhibitor (Ir-CPI)

Ir-CPI, a serine protease inhibitor originating from the salivary glands of the blood-feeding tick Ixodes ricinus, was first described in 2002 [68]. It selectively targets central elements of the contact activation pathway, including FXIIa, FXIa, and plasma kallikrein (PK). Ir-CPI was used in one of the first studies demonstrating the potential of FXII inhibition in preventing thrombosis (both venous and arterial) published nearly two decades ago by Decrem et al. [35] Importantly, these anticoagulant effects occurred without inducing bleeding or altering standard coagulation parameters.

As anticipated, intravenous delivery of Ir-CPI extends activated partial thromboplastin time (aPTT) while leaving prothrombin time (PT) largely unaffected, as confirmed by ex vivo clotting assays. In vivo studies have shown its effectiveness in both venous and arterial thrombosis models, such as ferric chloride (FeCl_3_)-induced thrombosis and stasis models [35].

During extracorporeal circulation in cardiac surgery in sheep, Ir-CPI has been shown to be as effective as UFH in preventing thrombosis [62]. No significant differences were observed in arterial or venous pressures or gas exchange parameters during the procedure. Postoperative macroscopic and histological evaluations revealed no notable inflammatory changes between Ir-CPI- and UFH-treated groups. Moreover, studies by Pireaux et al. demonstrated that Ir-CPI may have a superior safety profile compared to UFH. In a pig model undergoing liver biopsy, UFH-treated animals experienced significantly greater blood loss compared to those receiving Ir-CPI.

Furthermore, Ir-CPI appears to demonstrate neuroprotective effects in specific clinical scenarios. In preclinical studies, administration of Ir-CPI in mice significantly reduced neutrophil infiltration in hemorrhagic regions, decreased neuronal degeneration, and improved functional recovery [62]. The safety, tolerability, and efficacy of Ir-CPI in the treatment of spontaneous intracerebral hemorrhage (ICH) are currently being assessed in the ongoing phase IIa BIRCH trial [69]. The inhibitor is available under the trade name BIOX-101 and has been granted orphan drug designation for ICH in both the United States and the European Union, underscoring its therapeutic potential in an area lacking effective pharmacological interventions. As of 2025, preparation for a Phase IIb study is underway, positioning Ir-CPI as one of the most advanced FXII-pathway inhibitors in clinical development [70].

### 8.2. rHA-Infestin-4

rHA-Infestin-4, first reported by Hagedorn et al. in 2010 [48], is a recombinant fusion protein consisting of Infestin-4—a serine protease inhibitor from the midgut of *Triatoma infestans*—linked to recombinant human albumin (rHA) to extend its plasma half-life. This construct acts as a selective inhibitor of FXIIa.

In subsequent studies in animal models, rHA-Infestin-4 demonstrated the potential for effective anticoagulation with a favorable safety profile. In FeCl_3_-induced arterial injury models in mice and rats, prophylactic rHA-Infestin-4 completely abolished occlusive thrombus formation and maintained vessel patency throughout the observation period, whereas all control animals developed full arterial obstruction [48,51]. In carotid artery thrombosis models, rHA-Infestin-4 preserved arterial flow for at least 60 min after FeCl_3_ injury, and when thrombi did form, they remained small and unstable, preventing full occlusion [50]. The compound also exerted potent antithrombotic effects in venous, stasis-prompted, and foreign-surface-induced thrombosis, including arteriovenous shunt models in rats, rabbits, and mice, where it dose-dependently reduced clot weight and prevented occlusion triggered by highly thrombogenic artificial surfaces [51,60]. Importantly, these effects were achieved without compromising physiological hemostasis: rabbits treated with rHA-Infestin-4 exhibited normal hemostatic capacity, with only minimal prolongation of cuticle bleeding times and a modest 13% reduction in ex vivo FXa activity at higher doses, indicating limited off-target effects [51,60].

Beyond its antithrombotic properties, rHA-Infestin-4 demonstrated neuroprotective effects in models of ischemic stroke and silent brain ischemia. It significantly reduced infarct size, brain edema, and pathological fibrin deposition, and improved neurological scores and survival following transient MCA occlusion or embolic silent ischemia, without increasing hemorrhagic transformation [48,49,50]. Histological analyses further confirmed reduced microvascular thrombosis and better preservation of perfusion in the ischemic territory [49,50]. Collectively, these findings highlight rHA-Infestin-4 as one of the most potent preclinical FXIIa inhibitors, capable of preventing both arterial and venous thrombosis, mitigating foreign-surface–induced clotting, and conferring cerebrovascular protection while maintaining a favorable bleeding profile.

### 8.3. Monoclonal Antibodies

Monoclonal antibodies targeting FXII and FXIIa have emerged as highly selective experimental anticoagulants. Preclinical studies across multiple species, including mice, rabbits, and non-human primates, have evaluated several antibody-based FXII inhibitors with promising safety profiles. These agents collectively demonstrate the potential to reduce thrombus formation without inducing clinically relevant bleeding, especially in extracorporeal circulation and in long-term anticoagulation after stengraft implantation.

In a murine ECMO model, Xu et al. [64] demonstrated that a single-domain antibody targeting FXII (Nb-Fc) significantly reduced thrombus deposition on oxygenator membranes and systemic microvascular thrombi. In microfluidic human blood analysis, Nb-Fc also prevented collagen-induced fibrin formation and neutrophil adhesion/activation. Another promising agent, 3F7, a recombinant fully human monoclonal antibody against FXIIa, provided thromboprotection in a rabbit ECMO cardiopulmonary bypass model comparable to UFH. Importantly, 3F7 did not cause excessive bleeding from wound sites, which was a notable complication observed with standard UFH prophylaxis [61].

Subsequent research extended into non-human primate models. Administration of 5C12, a monoclonal antibody targeting the protease-containing domain of FXII, reduced platelet and fibrin deposition in extracorporeal membrane oxygenators—regardless of the presence or absence of low-dose UFH. Notably, the dose of 5C12 used did not significantly prolong bleeding time [63]. Treatment with monoclonal antibodies against the human FXII heavy chain and 14E11, which binds FXI interfering with fXI activation by fXIIa, reduced fibrin formation in collagen-coated vascular grafts inserted into arteriovenous shunts in baboons, and reduced fibrin and platelet accumulation downstream of the graft [58,59].

Although FXII inhibitors are not currently a viable alternative to antiplatelet agents or anticoagulants in the management of patients undergoing vascular stent implantation or treated with ECMO, and most evidence to date is limited to in vitro and animal models, these agents could have future applications. Specifically, they may benefit patients at high risk of bleeding or serve as adjuncts in individuals with a particularly high risk of stent thrombosis.

## 9. FXII in Hereditary Angioedema (HAE)

Hereditary Angioedema (HAE) is a rare, inherited autosomal dominant disorder, affecting approximately 1 in 50,000 people worldwide. Angioedema causes nonpitting swelling of subcutaneous and submucosal tissues, commonly affecting the face, extremities, and airway. That can be life-threatening, should the larynx be involved. Symptoms often emerge during adolescence; young children may remain asymptomatic despite genetic inheritance.

**Figure 2:**
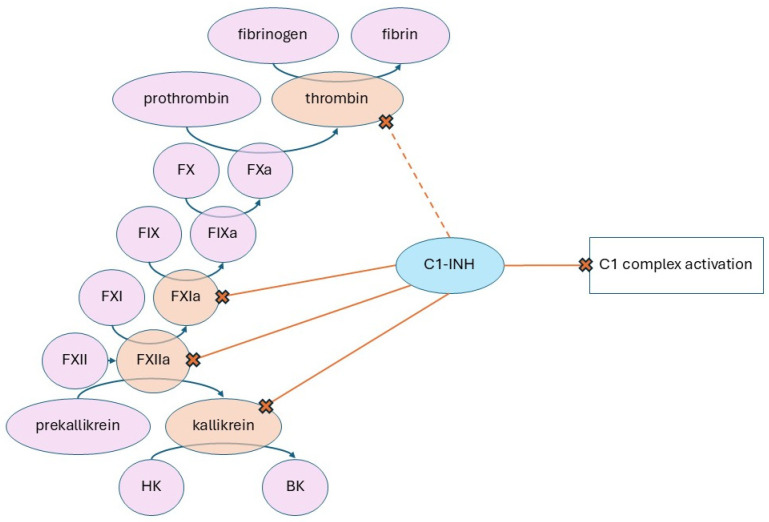
C1-esterase inhibitor (C1-INH) regulates both the complement and contact systems by inhibiting FXIa, FXIIa, and plasma kallikrein. Although C1-INH can inhibit thrombin, this interaction is kinetically slow and characterized by low affinity, making it of limited physiological relevance.

HAE is mostly caused by C1 esterase inhibitor (C1-INH) gene mutations resulting in its deficiency (type I) or impaired function (type II) [71,72]. HAE with normal C1-INH activity (HAE-nl-C1-INH; previously termed Type III) may result from FXII mutations or estrogen-dependent FXII activity enhancement. C1-INH inhibits FXIIa, FXIa, and plasma kallikrein, subsequently blocking high-molecular-weight kininogen (HMWK) to bradykinin (BK) conversion (Figure 2). Deficiency or dysfunction of C1-INH leads to uncontrolled kallikrein activity and excessive bradykinin production [73,74]. Elevated kallikrein also creates a positive feedback loop further promoting activation of FXII [75]. A gain-of-function missense mutation in the F12 gene, encoding factor XII, is one of several causes of HAE-nl-C1-INH leading to similar dysregulation as type I and II, resulting in increased vascular permeability and edema [72].

Diagnosis of HAE is based on C1-INH, C4, bradykinin levels and function. Acute HAE attacks are managed with infusions of C1-INH, receptor antagonists, and kallikrein inhibitors [76].

### 9.1. Garadacimab

Garadacimab is a fully human recombinant IgG4λ monoclonal antibody which targets the catalytic domain of activated factor XII (FXIIa) [77]. By blocking FXIIa, it prevents the activation of plasma kallikrein (PK) and the subsequent release of bradykinin (BK). Thus, garadacimab modulates a key pathway responsible for HAE symptoms. It has been proven to significantly reduce the frequency of HAE attacks in clinical studies perceiving an acceptable tolerance [78]. Garadacimab was approved by the European Medicines Agency (EMA) in February 2025 and by the U.S. Food and Drug Administration (FDA) in June 2025. It is administered subcutaneously once a month and is available as a pre-filled syringe or pen. However, it is important to note that there is limited data available on the use of garadacimab in HAE-nl-C1-INH due to alternative pathways that do not include FXII activation.

### 9.2. HAE and Thromboembolic Risk

C1-INH deficiency or dysfunction leads to dysregulated activation of factor XII, thereby providing a mechanistic link between hereditary angioedema (HAE) and an increased risk of deep vein thrombosis (DVT). Grover et al. demonstrated that plasma from patients with C1-INH-deficient HAE, compared with plasma from healthy controls, exhibits increased thrombin generation when coagulation is initiated via the intrinsic pathway using silica, but not when triggered through the extrinsic pathway with tissue factor. These findings suggest that contact system dysregulation contributes to hypercoagulability in HAE [79]. In animal models, C1-INH-deficient mice developed larger thrombi than wild-type controls and mice treated with C1-INH. Importantly, C1-INH-deficient mice showed a pronounced predisposition to venous, but not arterial, thrombosis. Elevated markers of thrombin generation were observed both at baseline and during angioedema attacks in animal models as well as in patients with HAE. Despite compelling experimental evidence supporting prothrombotic mechanisms in C1-INH deficiency, epidemiological evidence remains inconclusive. A Swedish registry study including 239 patients with hereditary angioedema (HAE) and 2383 matched controls reported increased risks of autoimmune and cardiovascular diseases in HAE, with a notably higher incidence of venous thromboembolism [80]. Nevertheless, clinically overt thrombotic events in HAE remain rare, with an estimated incidence of approximately 1 in 50,000. This discrepancy may reflect the low prevalence of HAE, unidentified physiological triggers of contact system activation, or insufficient magnitude and duration of thrombin surges during angioedema attacks. In selected clinical settings—such as acute illness or invasive procedures that may precipitate attacks—prophylactic anticoagulation may be considered [81].

## 10. Conclusions

Factor XII has transitioned from being regarded as a component of the intrinsic coagulation pathway of unknown significance to a key regulator at the interface of thrombosis, inflammation, fibrinolysis, and immunity. Evidence from genetic models, preclinical studies, and emerging clinical trials demonstrate that inhibition of FXII provides potent antithrombotic and anti-inflammatory effects without compromising physiological hemostasis. This unique safety profile is supposed to distinguish FXII inhibitors from currently used anticoagulants and position them as promising candidates for high-risk clinical scenarios.

Several classes of FXII inhibitors, including monoclonal antibodies, antisense oligonucleotides, peptides, and recombinant proteins, have shown efficacy in models of ischemic stroke, traumatic brain injury, extracorporeal circulation, and stent thrombosis, as well as in hereditary angioedema. Garadacimab was granted clinical approval, highlighting the translational potential of this therapeutic approach. Other agents, such as Ir-CPI or rHA-Infestin-4, continue to expand the preclinical and early clinical evidence base.

Nevertheless, a key limitation of the current evidence base is that most data on FXII inhibition originate from preclinical animal models, which, despite demonstrating consistent antithrombotic efficacy without increased bleeding risk, may not fully translate to human physiology. Species-specific differences in the relative contribution of the contact pathway to thrombus formation, as well as variations in inflammatory and complement system crosstalk, raise uncertainty regarding the magnitude of clinical benefit in humans. In addition, potential safety concerns remain insufficiently explored. While short-term studies suggest a favorable bleeding profile, the long-term effects of FXII inhibition, particularly immunogenicity associated with monoclonal antibodies, off-target interactions, and interference with complement activation, require careful evaluation in larger clinical populations. Importantly, FXII inhibition is being developed in parallel with factor XI-targeted strategies, which currently have a more advanced clinical evidence base. Although both approaches aim to dissociate thrombosis from hemostasis, they differ mechanistically and potentially in their clinical applicability. Therefore, a critical appraisal of the available evidence highlights that, while FXII inhibitors represent a promising therapeutic concept, their ultimate clinical role remains to be defined through robust human studies directly comparing efficacy, safety, and mechanistic profiles with established anticoagulant therapies.

The optimal positioning of FXII inhibitors within the broader anticoagulant landscape, whether as stand-alone therapies, adjuncts, or alternatives for patients at high bleeding risk, has yet to be defined. FXII inhibitors represent a promising frontier in antithrombotic therapy and immune modulation.

## Figures and Tables

**Table 2:** Presentation of current knowledge on the role of FXII in coagulation and the immune response associated with extracorporeal circuits and exposure to artificial materials in the form of stents and stent grafts.

Author	Year	Animal Models, Devices and Therapeutic Agents	Main Conclusion
Decrem Y et al. [35]	2009	○ Sprague-Dawley OFA rats ○ NMRI female mice	Ir-CPI selectively targets activated contact factors (FXIIa, FXIa, kallikrein) and prolongs aPTT in vitro. Its intravenous administration dose-dependently reduces venous thrombosis and impairs formation of occlusive arterial thrombi in rodents. Ir-CPI protects against collagen/epinephrine-induced thromboembolism in mice. It provides antithrombotic efficacy without increasing bleeding or altering global coagulation parameters.
Cheng Q et al. [58]	2010	○ FXII-deficient mice ○ antibody 14E11 binding FXI interfering with FXI activation by FXIIa	FXII deficiency confers greater antithrombotic protection than FXI or FIX deficiency in carotid artery injury models. The FXI-neutralizing antibody 14E11 prevents FeCl_3_-induced arterial occlusion, mimicking complete FXI deficiency in mice. Collagen-coated arteriovenous shunts. These findings identify FXIIa-driven FXI activation as a key amplifier of thrombosis in rodents and primates.
Yau JW et al. [55]	2014	○ New Zealand white rabbits ○ catheters implanted in the jugular vein	Compared with control, FXII and FXI antisense oligonucleotides treatment prolonged the time to catheter occlusion by 2.2- and 2.3-fold, respectively. Catheter thrombosis is triggered via the contact pathway.
Matafonov A et al. [59]	2014	○ male baboons (Papio anubis)	Monoclonal antibodies against the human FXII heavy chain reduced fibrin formation in collagen-coated vascular grafts inserted into arteriovenous shunts in baboons, and reduced fibrin and platelet accumulation downstream of the graft. These findings support a role for FXII in thrombus formation in primates.
Xu Y et al. [60]	2014	○ Sprague-Dawley male rats ○ New Zealand White male rabbits ○ rHA-Infestin-4	rHA-Infestin-4 dose-dependently reduces thrombus weight in arteriovenous shunt models in rats and rabbits. Bleeding times are minimally affected, indicating preserved hemostasis in both species. At 5 mg/kg in rabbits, rHA-Infestin-4 causes only a modest (~13%) reduction in ex vivo FXa activity, suggesting limited off-target effects.
Larsson M et al. [61]	2014	○ antibody 3F7 ○ wild-type mice ○ FXII-deficient mice ○ Rabbits	Recombinant fully human antibody binding catalytic site of human FXIIa (3F7) interfered with FXIIa-mediated coagulation and blocked carotid artery 10% FeCl3-induced thrombosis in mice and rabbits. 3F7 provided thromboprotection in ECMO as efficiently as heparin.
May F et al. [51]	2016	○ NMRI mice ○ C57Bl/6 mice ○ New Zealand White rabbits	rHA-Infestin-4 decreased occlusion rates in the mechanically-induced arterial (Folt’s) and the FeCl3-induced venous thrombosis model. It protected from FeCl3 -induced arterial thrombosis and from stasis-prompted venous thrombosis in rabbits. rHA-Infestin-4 prevented occlusion in the arterio-venous shunt model in mice and rabbits where thrombosis was induced via a foreign surface. Hemostatic capacity in rabbits was unaffected by rHA-Infestin-4.
Pireaux V [62]	2019	○ Ir-CPI vs. UFH ○ cardiopulmonary bypass with cardiac surgery in sheep ○ pig liver bleeding assays	Ir-CPI, a specific protein inhibitor of FXIIa, FXIa and kallikrein, was as efficient as UFH in preventing clot formation within the extracorporeal circuit and maintained physiological parameters during and post-surgery.
Wallisch M et al. [63]	2020	○ chronic exteriorized femoral arteriovenous (AV) shunt-bearing baboons ○ antibody 5C12	The FXII-neutralizing monoclonal antibody 5C12 reduces platelet deposition and fibrin accumulation in baboon extracorporeal membrane oxygenators, both with and without low-dose unfractionated heparin. Effective antithrombotic dosing does not prolong bleeding time, indicating preserved hemostasis.
Tweddell JS et al. [54]	2023	○ corn trypsin inhibitor ○ veno-arterial ECMO rabbit model	Inhibition of the contact pathway attenuates coagulation and inflammatory activation during ECMO. Tissue factor-driven thrombin generation may partially compensate for FXII inhibition under high inflammatory burden.
Xu P et al. [64]	2024	○ single-domain nanobody (Nb) fused to the Fc region of a human immunoglobulin (Nb-Fc) recognizing FXII ○ C57BL/6J mice	Inhibition of FXIIa formation attenuated arterial thrombosis in male mice without affecting hemostasis. In a mouse model of extracorporeal membrane oxygenation (ECMO), FXII inhibition or knockout reduced thrombus deposition on oxygenator membranes and systemic microvascular thrombi. In human blood microfluidic analysis, FXII inhibition prevented collagen-induced fibrin deposition and neutrophil adhesion/activation.
Keeling NM [65]	2024	○ Non-human primate model of nitinol stent-related thrombosis ○ monoclonal FXI and FXII antibodies	Function-blocking antibodies of FXII and FXI reduced markers of stent-induced thrombosis in vitro and ex vivo. Thrombosis markers under varied flow conditions.
Kharnaf M et al. [53]	2024	○ FXII deficient rats ○ veno-arterial ECMO rat model	FXII promotes thromboinflammatory response during ECMO. FXII deficiency reduces neutrophil infiltration and inflammatory markers despite anticoagulation. FXII may contribute to inflammation independently of thrombin generation.

ECMO—Extracorporeal Membrane Oxygenation; rHA-Infestin-4—complementary DNA of FXII inhibitor fused to recombinant human albumin; Ir-CPI—Ixodes ricinus contact phase inhibitor.

**Table 3:** Comparison of FXII inhibitors and their development stage.

Inhibitor/Class	Main Target(s)	Main (Pre) Clinical Setting(s)	Stage of Development (As of 2025)
Ir-CPI	FXIIa, FXIa, PK	venous and arterial thrombosis (FeCl_3_, stasis models); extracorporeal circulation; ICH models	phase IIa clinical trial (BIRCH) for spontaneous ICH
rHA-Infestin-4	selective FXIIa inhibitor	venous and arterial thrombosis foreign-surface thrombosis (AV shunts), ischemic stroke, silent brain ischemia	preclinical
monoclonal antibodies: 3F7, 5C12, Nb Fc, anti-FXII heavy chain	FXIIa, FXII heavy chain, FXIIa-mediated FXI activation	ECMO thromboprotection; stent and graft thrombosis; arterial injury models; thromboinflammation	preclinical
Garadacimab	fully human IgG4λ monoclonal antibody targeting FXIIa catalytic domain	hereditary angioedema (HAE); mechanistic studies in FXII inhibition, inflammation	approved in 2025 by EMA and FDA for HAE prophylaxis;

Ir-CPI—Ixodes ricinus Contact Phase Inhibitor; ICH—intracerebral hemorrhage; ECMO—Extracorporeal Membrane Oxygenation; HAE—hereditary angioedema; EMA—European Medicines Agency; FDA—Food and Drug Administration.

## Data Availability

No new data were created or analyzed in this study.
